# Maintaining the physical quality and digestibility of pellet feed through the use of plant-based pellet binder

**DOI:** 10.5455/javar.2024.k752

**Published:** 2024-03-31

**Authors:** Achmad Jaelani, Tintin Rostini, Muhammad Irwan Zakir, Sugiarti Sugiarti, Rayhana Fitryani

**Affiliations:** Department of Animal Husbandry, Agricultural Faculty, Kalimantan Islamic University, Banjarmasin, Indonesia

**Keywords:** Alabio duck, digestibility, physical quality, plant-based pellet binder

## Abstract

**Objectives:**

This study aimed to analyze the effects of the use of binders on the physical quality and digestibility of Alabio ducks (*Anas platyrinchos* Borneo).

**Materials and Methods:**

Pellet binders used tapioca meal (TM) (*Manihot utilissima*), sago meal (SM) (*Metroxylon sagu* Rottb.), and sweet potato meal (SPM) (*Ipomoea batatas*) pelleted feed. Laying Alabio ducks, around 120 birds, aged 20 weeks with an average body weight of 1,426 ± 113.5 gm, were used. A fully randomized design with 4 treatments and 15 repeats was used in this study. The variables measured include the physical quality and digestibility of pellet feed. Data analysis used a Fisher test. For the distinction between treatments, the Duncan multiple-range test was conducted.

**Results:**

The finding showed that the plant-based pellet binder had a natural effect on physical properties, including pellet durability index, moisture content, threshold power, stack density, and stack compacted density. The strength of the pellet binder is seen in the durability index of TM 98.12%, SM 97.64%, and SPM 97.35%, respectively. However, these variables did not differ significantly in terms of specific gravity and stack angle. Pellet binders considerably affect the consumption of feed and vary markedly in dry matter, organic matter, and metabolizable energy digestibility.

**Conclusion:**

Plant-based pellet binders influence the physical quality and digestibility of pelleted feed in Alabio ducks. TM can maintain physical quality and digestibility compared to SM and SPM as plant-based pellet binders.

## Introduction

Pelleted feed is a complete feed of nutrients because it comprises various feed ingredients generally given to poultry, including Alabio ducks (*Anas platyrinchos* Borneo). Commonly, pellet feed has an influential positive effect on duck performance. The advantages of pellets include feed intake, feed efficiency, improved digestibility, reduced material separation, and increased palatability [1].

Pelleted feed generally has strong physical properties, is not easily broken, and is completely nutritional and palatable. This, of course, really depends on the constituent materials. Adding pellet binders such as starch, gelatin, and hemicellulose extract enhances the pellet’s quality [2]. The presence of starch and its gelatinization are the most essential factors in producing the desired pellet quality.

Pellet binders strongly bond with all feed ingredients that make up pellets and can maintain pellet quality. Pellet binders can be made from various materials, including peptides, starch, and carboxymethyl cellulose [3]. The pellet binder peptides and carboxymethyl cellulose have been negatively affected besides the high price, and the material must be imported compared to local plant pellet binder [4]. Plant-based binders commonly used in pellet making include *Manihot utilissima*, *Metroxylon sagu* Rottb, *Ipomoea batatas*, and *Oryza sativa* L. Indica. This plant-based adhesive is commonly used as an adhesive in making pellets. Components that act as plant-based pellet binders from some carbohydrate sources are the presence of amylose and amylopectin [5]. The complete quality of pellets usually has a good integration of physical and biological factors. The physical quality of pellets includes specific gravity, threshold power, moisture content, durability index, stack angle, stack density, and stack compaction density. The biological properties include palatability, digestibility of organic matter(DOM), dry matter, and metabolizable energy.

The natural rate of pellet feed further strengthens that Alabio ducks also favor pellets besides being physically strong and having high digestibility in absorbing the nutritional content of the pellet feed. However, there are still ducks whose growth and production are not optimal, even though the feed given is in the form of pelleted feed. The first nutritional requirements to consider when creating a pellet diet are protein and energy, because they are the most expensive food ingredients and because of their effect on productivity [6].

Pelletized is still found to be not compact, not uniform, and easily destroyed at the time of manufacture and before being given to ducks. In particular, a feed composition of 40% influences the pelleted material’s physical quality [7]. Generally, in mixing pellet feed ingredients, it is less likely to consider the balance of plant-based pellet binders with other materials that impact the quality of pellets. According to the study’s findings by Syamsu [8], it was observed that the finest physical qualities were obtained when the pellet ratio included 5% tapioca flour, namely a 549 kg/m^3^ stack density and a 746 kg/m^3^ pile compaction density. Although the pellets produced are physically reasonable, there is still pelleted feed that ducks do not like and that has low nutrient digestibility. The novelty of this study is the use of plant-based pellet binders that can make pellets that not only have excellent physical condition but also have good digestibility for ducks.

Pellet feed production can alter the nutrient palatability and digestibility of the feed due to alterations in the chemical makeup of the meal, such as hydrolyzing proteins and gelatinizing starch. Animal species, feed shape, feed content, feeding space, ambient temperature, and livestock age all have an impact on how digestible a feed is [9].

Plant-based adhesives can compact pellet feed; however, the few pellets produced have poor physical properties. This is due to the binding power of plant-based pellet binders, which is not optimal. Pelleted feed must not only have good physical quality, but the most important thing is that pelleted feed must have high palatability and digestibility, so this is the importance of the research carried out. This study thoroughly tested the quality of plant-based pellet binders on the feed’s physical performance and digestibility in Alabio ducks.

## Materials and Methods

### Ethical approval

The ethics committee of the Research Ethics Commission of the Kalimantan Islamic University Banjarmasin Indonesian approved this study with protocol number 66/UNISKA-LP2M/E/II/2023.

### Birds and housing

A total of 120 laying Alabio ducks at the age of 20 weeks with an average body weight of 1,426 ± 113.5 gm were used. In a 60-pen cage (each cage contains 2 ducks), there were 15 replicate bird pens for each distinct treatment. Nipples, feeders, and sawdust for bedding were provided in each floor pen.

### Pellet manufacture

A counterpoise mixer weighing 900 kg (Hayes & Stolz Ind. Mfg. Co., Model TRDB63-0152) was used to combine the entire feed. The treatment is differentiated by the type of plant-based pellet binder according to the formulation. Having a capacity for 4.76 mm by 41.28 mm pellets, the Model 1012-2 HD One-Ton, 30-HP Pellet Mill was used to pelletize the mash diet, after 82°C conditioning.

### Test preparation

Utilizing the NHP 100 portable tester for pellet durability from Tek Pro Ltd., New Holmen, Norfolk, UK, this index measures the durability of pellets. A 100-gm sample of the 4.76-mm pellets was screened, after which it was put in the test chamber with perforation and shaken for 60 sec using compressed air [10]. The physical test of pellet feed refers to the method described by Sermyagina et al. [1]. Before the diet was prepared, all components were tested according to the Association of official agricultural chemists (AOACs) [11]. With the aid of near-infrared reflectance spectroscopy, the amino acid contents were identified. The Amylopectin/Amylose Assay Kit (K-AMYL 09/14) manufactured by Megazyme was used to conduct a colorimetric analysis to assess the dietary amylose/amylopectin ratio [12]. The entire collection technique of the indicator should be used to measure digestibility, as should the formula provided by Yu et al. [13]. Fecal organic matter is measured using the average measurement of fecal organic matter during the last 7 days of each study period directly. After 12 h, Alabio ducks are killed, and the large intestine is removed to obtain a stool sample to prevent contamination with urine [14]. A parabolic oxygen bomb calorimeter was used to calculate the gross energy (GE) value using the thermochemical benzoic acid as a reference. For measurements of digestibility of energy metabolizable, nitrogen-corrected metabolic energy (calculated by subtracting 8.22 kcal of nitrogen from the GE of excreta feed), and apparent metabolizable energy [13].

### Statistical analysis

SAS version 9.4’s PROC. The GLIMMIX technique was used to analyze the experiment’s data and examined as a randomized complete design using a statistical significance of *p* ≤ 0.05 established for analysis of variance, and the Duncan multirange test was employed to separate the means.

## Results and Discussion

Table 1 indicates that the moisture percentage of the adhesive with the addition of SM is higher than that of TM and SPM. The moisture content affects the stack’s density because a higher water content causes a drop in the pile’s density. Sweet potatoes had the highest water content (14.02%). In contrast, the plant-based pellet binders made from the baseline diet (BD), SM, and TM had lower water percentages than those of SPM (13.73%, 13.25%, and 12.86%, respectively). The water content in the material increases, and the pile’s density decreases since the material will expand and take up more space as a result [15].

Table 2 shows that the variance analysis revealed that using plant-based pellet binders such as TM and SPM had an appreciable effect (*p* ≤ 0.05) on the density of pellet feed stacks (569 and 586 kg/m^3^). The average values of BD and SM were 622 and 601 kg/m^3^. Among plant-based pellet binders, SM has the highest stack density value (601 kg/m^3^). The stack density is further impacted by the inclusion of adhesives, which purportedly possess binding properties and high starch content, and starch-producing plants can be used as adhesives in pelletizing. Materials with a low stack density (500 kg/m^3^) will cause flow to flow faster in the vertical direction [16]. The density of the stack, which has an impact on the density of the final pellet feed, is one of the problems with the automatic dosing of pelleted feed. It indicated that one specific component of the particle size or degree of exquisiteness of the feed also impacts the density of the feed pile [17]. The stack density level decreases with increasing feed particle size [18]. The average density of commercial feed pellets in pellet form is 0.7 gm/ml [19]. The stack’s density value indicates the material’s porosity, defined as the air gap between the material’s particles. The amount of water and foreign particles in the material is inversely related to the pile’s density value, so adding more water or foreign particles will make the pile less dense.

The density and compaction level of the material have a big impact on how much and precisely storage bins like silos, containers, and packaging can be filled. Knowing the stack compaction density value is essential because it can pack materials into still-vibrating containers. Due to the feeding process’s compression, there will be less space between particles, increasing the material’s weight per unit volume. The correlation between pile compaction density and stack density is highly significant when determining silo capacity and mixing materials. The stack’s density value will be affected by various compaction techniques. As compaction density increases, a pile of materials uses less space, and vice versa.

**Table 1.** Ingredients and nutrients composition of pellet diets.

Ingredients	Treatment			
	BD	SM	TM	SPM
Corn meal (%)	43	40	40	40
Rice bran (%)	16	14	14	14
Crude palm oil (%)	5	5	5	5
Soybean meal (%)	15	15	15	15
Fish meal (%)	8	8	8	8
*Moringa oliefera* meal (%)	5	5	5	5
Molasses (kg)	4	3	3	3
Sago meal (%)	0	6	0	0
Tapioca meal (%)	0	0	6	0
Sweet potato (%) Limestone (%)	0 1	0 1	0 1	6 1
Calcium hydrogen phosphate (%)	2	2	2	2
Vitamin and premix^1^ (%)	1	1	1	1
Total	100	100	100	100
Nutritional content*				
Metabolizable energy (kcal/kg)	2,712	2,756	2,745	2,764
Dry matter (%)	86.27	87.14	86.75	85.98
Crude protein (%)	17.31	17.24	17.87	17.62
Crude fiber (%)	3.40	4.06	3.88	4.12
Crude fat (%)	4.60	4.72	4.81	4.74
Ashes (%)	4.50	5.04	5.21	4.87
Calcium (%)	0.80	0.84	0.86	0.91
Total phosphorus (%)	0.65	0.69	0.71	0.65
Available phosphorus (%)	0.42	0.47	0.52	0.48
Chlorine (%)	0.77	0.92	0.78	0.83
Sodium (%)	0.36	0.38	0.41	0.35
Digestible lysine (%)	0.21	0.25	0.33	0.27
Digestible methionine (%)	1.33	1.04	1.38	1.32
Digestible threonine (%)	0.60	0.55	0.62	0.58
Methionine + cysteine (%)	0.98	0.87	0.92	0.86
Digestible tryptophan (%)	0.21	0.23	0.27	0.24
Zinc (mg)	73	75	73	76
Selenium (gm)	0.30	0.40	0.30	0.30

*Standard National Indonesia (SNI) 3010 for layer duck (2017)

Description: BD = Basal diet SM = Sago meal TM = Tapioca meal SP = Sweet potato meal.

Supplied per kilogram of diet: cyanocobalamin, 45 mg; folic acid, 1.6 mg; biotin, 0.25 mg; cholecalciferol, 3.3 IU; menadione 3.0 mg; nicotinic acid, 45 mg; pantothenic acid, 14 mg; pyridoxine, 3.4 mg; riboflavin, 6.8 mg; trans-retinol, 9.7 IU; DL-tocopheryl acetate 52 IU’ thiamin, 2.2 IU; ME was a calculated value ME = (1.01×DE–0.45) + 0.0046 **×** (EE–3).

**Table 2.** Physical quality of pelletized feed with a variety of different plant-based pellet binders (*n =* 120).

Variables	Treatments			
	BD	SM	TM	SPM
Moisture content (%)	13.73^b^	13.25^ab^	12.86^a^	14.02^c^
Stack density (kg/m^3^)	622^b^	601^b^	569^a^	586^a^
Stack compaction density (kg/m^3^)	684^a^	652^b^	673^b^	664^b^
Stack angle (^o^)	41.62	41.85	42.48	41.94
Specific gravity	1.273	1.261	1.267	1.290
Threshold power (m/sec)	32.84^a^	33.91^b^	32.19^a^	35.05^bc^
Durability index (%)	96.42^a^	97.6^4^b	98.1^2^c	97.3^5^b

Description : BD = Basal diet SM = Sago meal TM = Tapioca meal SPM = Sweet potato meal

Numbers followed by different letter superscripts showed different results *p* ≤ 0.05.

According to variance analysis, adhesives made from SPM, TM, and SM significantly impact the pile’s angle (*p* ≤ 0.05). TM (42.48°C) exhibits a difference and has a broader stack angle than BD (41.62°C), SM (41.85°C), and SPM (41.97°C), which do not. The pellets treated with TM vegetable adhesive had the most significant size. The angular value of the pile will decrease with increased pellet size. The water content also affects the angle of the pile in addition to particle size; the higher the water content, the higher the pile’s corner value [20]. The presence of starch in the process of water and heat penetration simultaneously into the starch granule causes granule development and has an impact on pellet size [21]. The stack angle is broken down into various categories, including 20°C–30°C for materials that flow effortlessly, 30°C–38°C for materials that flow easily, and materials that are medium range from 38°C to 45°C; difficult-to-flow materials range from 45°C to 55°C [22]. This demonstrates how using plant-based adhesives alters the pile’s angular value. However, in general, the pile in this study’s angular value is still excellent because it is less than 50°C.

Vegetable adhesives do affect the specific gravity of pellet feed. All plant-pellet binder treatments have relatively statistically similar specific gravity values. This is thought to be because the homogeneity of the plant-based adhesive particles is more stable in the pellet ration mixture, thus making the specific gravity of the pellet relatively the same. Boltz et al. [23] explain that specific gravity and particle size are responsible for the homogeneity of particle dispersion and its stability in a feed mixture. Judging from the threshold power value, it turns out that the SPM treatment is the highest among BD, SM, and TM treatments. It turns out that the SPM threshold power value of 35.05 m/sec has the highest specific gravity of 1.290. Threshold power positively correlates with specific gravity [24].

Durability index measurements are used to estimate the resulting pellet’s resistance to effect, drop, or free crushing during storage or transportation. The impact value of pellet feed using SM, TM, and SPM vegetable adhesives is higher than that of those that do not use vegetable adhesives (BD). The pellet binder used shows that TM has the highest durability index of 98.12%. Feedstock with smaller particle sizes produces pellets of high quality. The impact resistance strength of 98.80%–99.40% depends on the particle size of the raw material used to make pellets, and the pellet durability index requirement is 80% [10]. Lyon et al. [25] assert that the uniformity of the particle sizes generated by hammer mills and roller mills with varying screen and roller spacing affects the longevity of the pellet index. Additionally, a high fiber content in the material might make the pellet break easily, resulting in a low durability index rating. Agrawal et al. [26] further stated that the diameter of the pellet is another element that may impact its durability; compared to pellets with a 6 mm diameter, those with a diameter of 3 mm are simpler to break.

The highest amylopectin content is found in SM, compared to TM and SPM. If we calculate the ratio of amylose to amylopectin statistically, SPM and TM do not differ but differ for SM and BD. The highest ratio value is SPM, and the lowest is BD. Results are shown in Table 3. The amylose/amylopectin ratio affects starch’s gelatinization and recrystallization properties. According to Zhao [27], the ratios of high and moderate levels of amylose to amylopectin are 0.60 and 0.47, respectively, and low levels are 0.23. Starch that contains a high ratio of amylose and amylopectin is both adhesive and wet. A higher ratio of amylose to amylopectin content results in high durability values. Amylose and amylopectin recrystallization are entirely different processes. Recrystallized amylopectin initially melts at about 40°C, while recrystallized amylose melts at temperatures above 120°C [28]. Recrystallized amylose is considered a significant source of feed.

The palatability of feed can be seen by how much feed is consumed. Judging from feed consumption, it shows that the use of vegetable adhesives has a positive effect on feed consumption. Alabio ducks that are laid and fed with TM and SPM feed consume more feed than those fed with BD and SM. This shows that pellet feeds with SPM and TM are more attractive to Alabio ducks. The process of starch gelatinization produces a flavor that can increase feed consumption. This follows the statement of Taylor et al. [29], which states that animal characteristics (body weight and age), feed digestion rate, feed quality, and palatability are some of the elements that affect feed consumption. Yang et al. [30] add that feed digestibility and feed digestion rate affect ration consumption; high digestibility and rapid digestion rate will increase ration consumption.

**Table 3.** Starch content, palatability, and digestibility of pelleted feed with a variety of different plant-pellet binder (*n =* 120).

Parameters	Treatments			
	BD	SM	TM	SPM
Amylose (%)	5.24^a^	7.63^b^	8.08^bc^	8.32^c^
Amylopectin (%)	16.68^b^	22.46^b^	19.22^a^	18.47^ab^
Amylose-amylopectin ratio	0,31^a^	0.34^a^	0.42^b^	0.45^b^
Feed consumption (gm/bird/day)	142.18^a^	152. 59^b^	156.24^b^	148.04^ab^
Digestibility of dry matter (%)	76.18^a^	78.34^b^	78,55^b^	77.46^ab^
Digestibility of organic matter (%)	75,26^a^	76,17^b^	77,43^c^	76.22^b^
Digestibility of Energy Metabolizable (%)	78.6^a^	79.44^a^	84.62^b^	87.3^b^

Description: BD = Basal diet SM = Sago meal TM = Tapioca meal SP = Sweet potato meal

Numbers followed by different letter superscripts showed different results *p* ≤ 0.05.

The amount of feed that is absorbed by the organism can be determined by measuring the dry matter content and examining the dry matter content of the ration and waste. The use of plant-based pellet binders affects the digestibility of dry matter. SM and TM vegetable adhesives have a higher digestibility value than BD and SPM. This indicates that SM and TM are quite effective as plant-based pellet binders because they produce better digestibility of dry matter than others. This may be because SM and TM starches, which digest quickly, are more easily oxidized, whereas SPM starches, which digest slowly, are more likely to promote de novo lipogenesis. The use of plant-based pellet binders affects the DOM. According to the statement of Volpato et al. [31], the contents and qualities of feed, especially feed used to increase the compactness of pelleted feed, considerably affect the organic matter’s capacity to be digested. All organic feed pellets with plant-based pellet binders have a higher digestibility than BD. For plant-based pellet binders, SM and SPM have a DOM that isn’t different but differs markedly compared to TM. The dry matter’s digestibility value and the organic matter’s digestibility for each vegetable adhesive do not differ much. Organic matter’s and dry matter’s digestibility of plant-based pellet binder materials is the highest obtained for TM treatment. According to Wang et al. [32], there is a positive correlation between the dry matter’s and organic matter’s digestibility in good-quality poultry feed. This can be seen from the use of TM in plant-based pellet binders in laying Alabio duck.

The metabolic yield of feed released in the form of excreta will be lower than the metabolic energy of feed because it has undergone the digestive process. Considering the study’s outcomes, it was concluded that pelleted feed using plant-pellet binder has better energy digestibility compared to BD. The highest metabolic energy digestibility is obtained in TM treatment compared to SM and SPM. The highest results are also seen with the TM treatment of feed consumption value. This indicates that vegetable adhesives made from TM can better bind other materials, resulting in more homogenous, compact, and palatable pellet component ingredients. The digestibility of metabolic energy will be influenced by the palatability value [31].

The use of a plant-based pellet binder that has the best physical properties known, 6% tapioca flour, is described, but the digestibility value is only based on the digestibility of metabolic energy, organic matter, and dry matter; the digestibility of amino acids and high feed rates of amylopectin are not described.

## Conclusion

Plant-based pellet binders significantly affect the physical properties and digestibility of pelleted feed in laying Alabio ducks. Tapioca plant-based pellet binders can maintain physical quality and digestibility compared to sago plant-based binders and sweet potato meal. It is recommended in the process of manufacturing pelleted feed containing 6% tapioca meal to strengthen its physical properties and digestibility in laying Alabio ducks.

## List of Abbreviations

AOAC, Association of Official Agricultural Chemists; DOM, Digestibility organic matter; SM, Sago meal; SPM, Sweet potato meal; TM, Tapioca meal
